# Intraspecific virulence of entomopathogenic nematodes against the pests *Frankliniella occidentalis* (Thysanoptera: Thripidae) and *Tuta absoluta* (Lepidoptera: Gelechiidae)

**DOI:** 10.21307/jofnem-2021-102

**Published:** 2021-12-14

**Authors:** Raquel Campos-Herrera, Ignacio Vicente-Díez, Magda Galeano, Maryam Chelkha, María del Mar González-Trujillo, Miguel Puelles, David Labarga, Alicia Pou, Javier Calvo, José Eduardo Belda

**Affiliations:** 1 Instituto de Ciencias de la Vid y del Vino (CSIC, Universidad de La Rioja, Gobierno de La Rioja) Finca La Grajera Ctra. Burgos Km. 6 Salida 13 Lo-20, Logroño 26007, Spain; 2 R&D Department of Koppert España, S.L. Paraje Piedra Rodada, 470, Vícar, Almería 04738, Spain; 3 Research Team “Lombricidae, Improving Soil Productivity and Environment” (LAPSE), Ecole Normale Supérieure (E.N.S.), Centre Eau, Ressources Naturelles, Environnement et Développement Durable (CERNE2D), Mohammed V University, Avenue Mohamed Bel Hassan El Ouazzani, BP: 5118, Takaddoum – Rabat, Morocco

**Keywords:** *Heterorhabditis*, *Steinernema*, Tomato, Aerial insect-pests

## Abstract

Entomopathogenic nematodes (EPN) are excellent biocontrol agents against various insect pests. Novel biotechnological approaches can enhance their utility against insects above-ground, opening a new venue for selecting superior EPN against certain insects. We hypothesize that different populations of the same species but from different origins (habitat, ecoregion) will differ in their virulence. This study aimed to evaluate the virulence of various EPN populations against two pests of worldwide incidence and damage to high value crops: *Frankliniella occidentalis* (Thysanoptera: Thripidae) and *Tuta absoluta* (Lepidoptera: Gelechiidae). We tested 10 EPN populations belonging to three EPN species: *Heterorhabditis bacteriophora* (Koppert, MG-618b, AM-203, RM-102), *Steinernema feltiae* (Koppert, RS-5, AM-25, RM-107), and *Steinernema carpocapsae* (Koppert, MG-596a). Each EPN population was tested at two concentrations. *Frankliniella occidentalis* was tested at 160 and 80 IJs/cm^2^ and *T. absoluta* at 21 and 4 IJs/cm^2^. Control treatments followed the same experimental procedure but only adding distilled water. Overall, whenever different, higher IJs concentration resulted in lower adult emergence, higher larval mortality, and shorter time to kill the insects. Considering the low concentration, *S. feltiae* provided the best results for both insects and instars investigated, while *H. bacteriophora* and *S. carpocapsae* required a high concentration to reach similar or slightly better results. Differences among populations of each of the species were detected, but only the native populations of *H. bacteriophora* populations showed consistently higher control values against both insects/instar compared with the commercial one. Differences among *S. feltiae* and *S. carpocapsae* populations depended on the IJs concentration, insect, and instar. We consider *S. feltiae* a very promising species for their application against *F. occidentalis* and *T. absoluta*, with the Koppert population as the most consistent among the populations tested. Specific EPN-populations of *S. carpocapsae* and *H. bacteriophora* were good candidates against certain instar/insects at high concentrations. This study emphasized the importance of intraspecific variability for EPN virulence.

Entomopathogenic nematodes (EPNs) in the genera *Steinernema* and *Heterorhabditis* are excellent biocontrol agents against many arthropod species (Campos-Herrera, 2015; Lacey et al., 2015). They are naturally distributed in the soil in the form of infective juvenile (IJ), which has to survive while searching for a host (Kaya et al., 2006). Once the victim is located, the IJs penetrate and release the mutualistic bacteria of the genera *Xenorhabdus* for steinernematids and *Photorhabdus* for heterorhbaditids (Adams et al., 2006). The arthropod is killed within 2–5 days (Dillman et al., 2012; Stock, 2015). The nematode and its bacterial partner reproduce inside the cadaver until the food is depleted and excretory products are excessive. Then, the nematodes develop the IJ form, acquire some bacteria and emerge from the cadaver to initiate the life cycle again.

The EPN species differ for traits related to the ability to survive in the environment (i.e. extreme conditions of temperature, humidity, etc.), to overcome predation or competition for resources (i.e., escape from nematophagous fungi), search for a host (i.e. foraging behavior cruiser vs. ambusher), kill (virulence associated with the nematode and the associated bacteria), or reproduce (Campbell et al., 2003; Grewal et al., 2006; Griffin, 2015). However, less is known about the intraspecific variability of isolates belonging to the same species but from different origins (habitat, ecoregion). As a result of environmental pressures, intraspecific variability might occur in critical traits associated with survival, the search of the host, virulence, etc. For example, intraspecific variability in parameters related to reproductive capability and life cycles, virulence against the agricultural pests *Ceratitis capitata* (Diptera: Tephritidae) and *Spodoptera littoralis* (Lepidoptera: Noctuidae), and the physiology and molecular profile of their associated bacteria was described among several isolates of *S. feltiae* from various habitats from La Rioja (Northern Spain) (Campos-Herrera et al., 2007, 2008, 2009; Campos-Herrera and Gutierrez, 2009, 2014). Another example of intraspecific variability was provided by Bruno et al. (2020). They evaluated the virulence of 40 EPN isolates belonging to five different species against *Diabrotica virgifera virgifera* (Coleoptera: Chrysomelidae) and *Diabrotica balteata* (Coleoptera: Chrysomelidae). They observed that while all the isolates infected non-sequestering benzoxazinoid-based defenses larvae *D. balteata*, the infectivity varied for the sequestering *D. virgifera virgifera*. Also, they detected some intraspecific variability in the impact of the benzoxazinoids on the infectivity of *H. bacteriophora* populations, although most of them were resistant. Finally, a recent study by Levy et al. (2020) revealed intraspecific variability on the tolerance to heat and desiccation. By using several bioassays, Levy et al. (2020) defined which populations performed better under stressing conditions, indenturing key genes involved in the tolerant mechanisms. All these examples illustrates the relevance of including intraspecific characterization of EPN when screening for certain abilities.

The insects *Frankliniella occidentalis* (Thysanoptera: Thripidae), and *Tuta absoluta* (Lepidoptera: Gelechiidae) are among the most devastating aerial pests worldwide (Siguna, 2007; Urbaneja et al., 2013; Biondi et al., 2018; Mansour et al., 2018; Dlamini et al., 2019a). *Frankliniella occidentalis* is one of the most critical thrips species associated with many greenhouse crops. Besides cosmetic injury that reduces value, they transmit viral diseases such as tomato spotted wilt virus (Moritz et al., 2004). The cryptic behavior, quick reproduction, and ability to develop resistance to insecticides make them a challenging pest (Gouli et al., 2008). Similarly, *T. absoluta* is the most serious insect pest of tomato grown in greenhouses or in the field, widely distributed in America, Europe, Africa, the Middle East, and Asia (Urbaneja et al., 2013; Biondi et al., 2018; Mansour et al., 2018). Females lay eggs on the aerial part of the tomato plant, and the larvae enter leaves, stems, and fruits producing the damage as galleries or mines that increase with the size of the larvae. Depending on the severity of the damage, the insect can reduce fruit quality, final yield, or kill the plant. The mines also promote the entrance of opportunistic pathogens (Biondi et al., 2018; Mansour et al., 2018).

The use of chemical insecticides is the most frequent control measurement for both pests, whose cryptic nature promotes extensive use that increases resistance development (Zhao et al., 1995; Gao et al., 2012; Urbaneja et al., 2012; Terzidis et al., 2014; Biondi et al., 2018; Mansour et al., 2018). The global concern about air, soil, and water pollution by, their impact on non-target organisms and potential impact on human health are promoting a shift toward strategies that provide efficient and non-polluting pest management tools such as entomopathogens, parasitoids and/or predators (Brødsgaard, 2004; Shipp and Ramakers, 2004; Messelink and Janssen, 2014; Lee et al., 2017; Damalas and Koutroubas, 2018; Mansour and Biondi, 2020). Whereas the cryptic nature of these two pests decreases the efficiency of some biocontrol agents (Dlamini et al., 2020; Ben Husin and Port, 2021; Dlamini et al., 2020), the active searching capacity of EPN can overcome some of these limitations (Griffin, 2015). Indeed, several studies have explored the efficacy of EPN against both pests with various successes. The soil-dwelling stages of *F. occidentalis* proved highly susceptible to various EPN species (Chyzik et al., 1996; Buitenhuis and Shipp, 2005; Ebssa et al., 2006; Dlamini et al., 2019b), which performed less effectively against larvae in aerial parts (Buitenhuis and Shipp, 2005). Indeed, the species *Steinernema feltiae* is widely suggested to manage *F. occidentalis* (Premachandra et al., 2003; Ebssa et al., 2004). Similarly, EPN can kill *T. absoluta* larvae and pupae (Batalla-Carrera et al., 2010; van Damme et al., 2013; Mutegi et al., 2017; Dlamini et al., 2020). The current development of novel formulation and application systems (Shapiro-Ilan and Dolinski, 2015) is expanding the persistence and activity of the EPN against aerial pests. Because populations of the same species from different origins (habitat, ecoregion) often differ in their virulence due to particular life-history traits selections, populations screening of suitable populations to identify superior lines ability can increase their performance in biological control programs. This study aimed to evaluate the virulence of various populations of three EPN species from natural or commercial origins against *F. occidentalis* and *T. absoluta.* Specifically, we investigated EPN (i) suppression of pupa emergence of both insects, and (ii) ability and time to kill the last instar larva of *T. absoluta*. We explored the intraspecific differences for each of the three EPN species using two concentrations of nematodes.

## Material and methods

### Insects, nematodes, plants, and substrates

Both insects, *F. occidentalis* and *T. absoluta* were provided by the R&D Department of Koppert (Spain) in the appropriate developmental instar, in scheduled and coordinated shipments. In all the cases, we ensured that in no case more than 2 days passed from their arrival until their experimentation, storing them under controlled conditions (60% Relative Humidity, RH, 22°C and a photoperiod of 16 hr of light -L- and 8 hr of darkness -D-) in isolated mesh-cages to avoid escapes.

A total of 10 EPN populations from three species (*S. feltiae, S. carpocapsae*, and *H. bacteriophora*) were investigated, comprising seven native and three commercial ones (Table 1). All nematodes were reproduced in the insect host *Galleria mellonella* (Lepidoptera: Pyralidae) (Woodring and Kaya, 1988). The infective juveniles (IJs) were collected in containers with mineral water and stored at 14°C. In any case, the nematodes were used in the studies within 15 to 20 days of emergence.

**Table 1.** Populations and species of nematodes of the genera *Steinernema* and *Heterorhabditis* investigated in this study

Entomopathogenic nematode species	Population	Origin	ITS sequence (GenBank accession number)
*S. feltiae*	RS-5	Switzerland	KJ938569
	AM-25	Portugal	MG551674
	RM-107	Spain	MW480131
	Koppert	Commercial	-
*S. carpocapsae*	MG-596a	Switzerland	MZ914694
	Koppert	Commercial	-
*H. bacteriophora*	MG-618b	Switzerland	MZ914695
	AM-203	Portugal	MG551676
	RM-102	Spain	MW480132
	Koppert	Commercial	-

For the experiments with *T. absoluta*, tomato plants *Solanum lycopersicum* var. moneymaker of 5 to 6 true leaves were maintained in sterilized greenhouse plant substrate in 1 L containers under controlled conditions (60% RH, 22°C and 16L: 8D). Finally, as substrate in the *F. occidentalis* experiments, we used pure mineral sand (Vale do Lobo, Loulé, Portugal). Before the experiments, the sand was washed several times with running water, autoclaved for 1  h (two times in two consecutive days as suggested by Elhady et al., 2018), oven-dried at 40°C with ventilation, and stored in laboratory conditions for at least a week before use (Chiriboga et al., 2017).

### Virulence study against pupae of Frankliniella occidentalis

We followed the procedures described by Ebssa et al. (2001) slightly modified. Briefly, the experimental unit was a mini-container with a transparent lid with hermetic closure to avoid escapes (2.5 cm diam. X 1.5 cm height). Each container was filled with 1 g of sterile sand. The final volume per mini-container was 100 μL (10% moisture w/v). With the aid of an A000 brush, 10 late-stage *F. occidentalis* larvae were individually caught for each mini-container, the corresponding treatments applied, and the containers were stored in controlled environment chamber (60% RH 22°C and 16L: 8D). Pollen was added to each mini-container 24 h postinoculation. The treatments included control (100 mL distilled water), and application of 100 μL suspension of 500 IJs (160 IJs/cm^2^) or of 250 IJs (80  IJs/cm^2^). Experiments were conducted by species, including in each trial all the corresponding populations. Treatments were replicated 5 times. The presence of adults was checked every 2 to 3 days post-inoculation, determining the adults’ emergence after 12 days (Campos-Herrera and Gutierrez, 2009). The experiment was performed twice, with new insects, nematodes, and substrate preparation.

### Virulence study against Tuta absoluta

The experimental unit was a 5.5 cm diam. Petri dish with one Whatman filter paper no. 1, a piece of 1 cm^3^ cotton, and two tomato leaves. In each dish, 8 last stage larvae of *T. absoluta* were included and 500 μL of distilled water was added to soak the cotton and partially moisten the filter paper. When studying the impact of EPN on pupae, the dishes were closed with parafilm and observed after 24 to 48 hr to verify the pupal status and the formation of galleries. When studying EPN efficacy against the last instar, nematodes were applied 6 hr after larval disposal in the dishes to ensure gallery formation but no pupation. In both studies, nematodes were applied in a final volume of 400 μL, with distilled water for controls. All dishes were stored in a controlled environment chamber (60% RH 22°C and 16L: 8D). In both, the pupa and last instar larva studies, the design and replication of the treatments were as for *F. occidentalis,* but with the concentrations of 500 IJs (21 IJs/cm^2^) and concentration of 100 IJs (4 IJs/cm^2^) applied in 400 μL. In the study against the pupal stage, the presence of adults was checked every 2 to 3 days post-inoculation for 12 days (Campos-Herrera and Gutierrez, 2009). In the study of the last instar larva, the mortality was checked daily until 5 days post-exposure, considering larval mortality and the number of days to the death as variables. The experiments were performed twice, with new insects, nematodes, and plants preparation.

### Statistical analyses

We calculated the percentage of adult emergence and larval mortality. Before statistical analysis, percentage values were arcsine transformed. After checking the statistical similarity of the results, data from different trials were combined (data not shown). For each of the experiments, we ran generalized linear models (GLM, *P* < 0.05) for the analysis of the percentage of the variable of adult emergence, percentage of larval mortality, and the number of days needed to die. We explore the intraspecific variability considering the factors “population” for each species (five levels, comprising control and the four populations for *S. feltiae* and *H. bacteriophora*, and three levels, control and two populations for *S. carpocapsae*), concentrations (two levels, high and low as described above), and their interactions. Besides, we performed an individual one-way ANOVA and Tukey test (*P* < 0.05) for each species (interspecific analysis) or population (interspecific analysis) and concentration to disentangle the control potential against both insect pests in the corresponding instar. Finally, the Student’s *t* test (*P* < 0.05) investigated the differences between the two concentrations in each of the species/populations for all the variables. We performed all the analyses with SPSS 25.0 (SPSS Statistics, SPSS Inc., Chicago, IL, USA). We used least-square means ± S. E. as descriptive statistics.

## Results

### Virulence study against pupae of Frankliniella occidentalis

**Figure 1:**
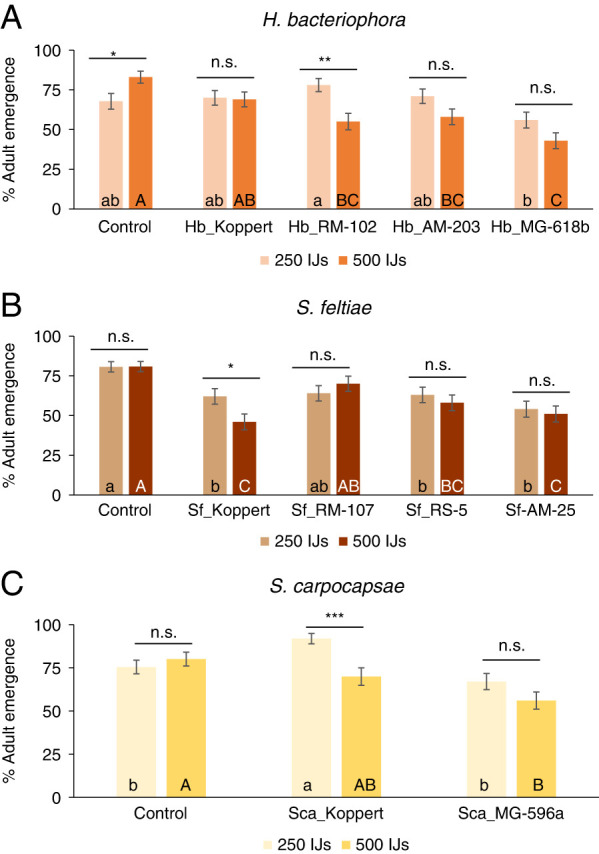
Intraspecific virulence of various populations from three entomopathogenic nematodes (EPN) species (*Steinernema feltiae*, *Steinernema carpocapase*, and *Heterorhabditis bacteriophora*) against *Frankliniella occidentalis.* (**A**) Percentage of *F. occidentalis* adult emergence after the application of four *H. bacteriophora* populations. (**B**) Percentage of *F. occidentalis* adult emergence after the application of four *S. feltiae* populations. (**C**) Percentage of *F. occidentalis* adult emergence after the application of two *S. carpocapsae* populations. Each population was applied in two concentrations: 500 JIs (160/cm^2^) and 250 JIs (80 IJs/cm^2^). Different small letters indicate significant differences in Tukey’s test (HSD) for the low concentration analysis. Different capital letters indicate significant differences in Tukey’s test (HSD). Above each species, asterisks indicate significant differences within treatment t student comparisons at *P < 0.05, **P < 0.01, ***P < 0.001, and n.s., not significant. Values are least-square means ± SE.

Percentage emergence of adults was significant for all the factors (populations and concentration) and their interactions except for *S. feltiae* (population and interaction) (Supplementary data Table S1, Fig. 1). We analyzed both factors individually to disentangle for concentration or population effects. Differences among populations were observed for both concentrations for each of the species (Supplementary data Table S2), while differences between concentrations for the same population were only observed in one population of each species (Fig. 1, Supplementary data Table S3). In detail, in the analysis of *H. bacteriophora* populations, differences with the emergence observed in the control were only observed for the native populations (Hb_RM-102, Hb_AM-203, and Hb_MG-618b), that reached 58 to 43% emergence (Fig. 1A). In the case of the *S. feltiae* populations, all the populations except Sf_RM-107 registered lower adult emergence than the control for both concentrations tested, with Sf_Koppert registering 46% adult emergence, the lowest value of all of the *S. feltiae* populations (Fig. 1B). Finally, for the *S. carpocapsae* intraspecific analysis, only Sca_MG-596a applied at the high concentration resulted significantly lower than the control emergence (Fig. 1C, Table S2).

**Table S1.** Statistical analysis of the effect of two factors (infective juvenile concentration and EPN population) and their interactions (GLM, *P* < 0.05) for the variables percentage of adult emercence for *Frankliniella occidentalis* and *Tuta absoluta,* and the larval mortality percentage and number of days to kill the last instar larvae of *T. absoluta*. Analysis performed considering each of the popualtions of the three species *Heterorhabditis bacteriophora* (Hb)*, Steinernema feltiae* (Sf), and *S. carpocapsae* (Sc).

Insect species	Variable	EPN species or populations	Concentration (C)	Population (P)	Interaction C*P
*F. occidentalis*	% Adult emercence	Hb populations	***F*** **= 5.474,** ***P** ***= 0.020**	***F*** **= 8.357,** ***P** ***< 0.001**	***F*** **= 4.757,** ***P** ***= 0.001**
		Sf populations	***F*** = 1.564, *P* = 0.211	***F*** **= 15.374,** ***P** ***< 0.001**	*F* = 1.533, *P* = 0.190
		Sc populations	***F*** **= 6.951,** ***P** ***= 0.009**	***F*** **= 11.103,** ***P** ***< 0.001**	***F*** **= 4.636,** ***P** ***= 0.010**
*T. absoluta*	% Adult emercence	Hb populations	***F*** **= 9.885,** ***P** ***= 0.002**	***F*** **= 15.925,** ***P** ***< 0.001**	***F*** **= 4.067,** ***P** ***= 0.003**
		Sf populations	*F* = 0.963, *P* = 0.327	**F = 7.794,** ***P** ***< 0.001**	*F* = 0.496, *P* = 0.739
		Sc populations	***F*** **= 10.655,** ***P** ***= 0.001**	***F*** **= 7.080,** ***P** ***= 0.001**	*F* = 2.178, *P* = 0.114
	% Larval mortality	Hb populations	***F*** **= 14.531,** ***P** ***< 0.001**	***F*** **= 90.407,** ***P** ***< 0.001**	*F* = 1.468, *P* = 0.210
		Sf populations	***F*** **= 7.649,** ***P** ***= 0.006**	***F*** **= 1609.766,** ***P** ***< 0.001**	*F* = 1.278, *P* = 0.277
		Sc populations	***F*** **= 18.917,** ***P** ***< 0.001**	***F*** **= 340.796,** ***P** ***< 0.001**	*F* = 2.989, *P* = 0.051
	No. Days to kill	Hb populations	***F*** **= 9.455,** ***P** ***= 0.002**	***F*** **= 4.973,** ***P** ***= 0.001**	***F*** **= 3.367,** ***P** ***= 0.010**
		Sf populations	***F*** = 1.686, *P* = 0.195	***F*** **= 39.515,** ***P** ***< 0.001**	***F*** **= 11.649,** ***P** ***< 0.001**
		Sc populations	***F*** **= 5.179,** ***P** ***= 0.023**	***F*** **= 11.109,** ***P** ***< 0.001**	***F*** **= 7.471,** ***P** ***= 0.001**

**Table S2.** Statistical analysis of the effect of infective juvenile (IJs) concentration (ANOVA, *P* < 0.05) for the variables percentage of adult emercence for *Frankliniella occidentalis* and *Tuta absoluta,* and the larval mortality percentage and number of days to kill the last instar larvae of *T. absoluta*. Analysis performed considering the popualtions of each the three species *Heterorhabditis bacteriophora* (Hb)*, Steinernema feltiae* (Sf), and *S. carpocapsae* (Sc).

Insect species	Variable	EPN populations	500 IJs	250 IJs (*F. occidentalis*) 100 IJs (*T. absoluta*)
*F. occidentalis*	% Adult emercence	Hb populations	***F***_**4,499**_ **= 10.192,** ***P** ***< 0.001**	***F***_**4,489**_ **= 3.006,** ***P** ***= 0.018**
		Sf populations	***F***_**4,550**_ **= 11.148,** ***P** ***< 0.001**	***F***_**4,549**_ **= 5.714,** ***P** ***< 0.001**
		Sc populations	***F***_**2,292**_ **= 6.766,** ***P** ***= 0.001**	***F***_**2,303**_ **= 8.734,** ***P** ***< 0.001**
*T. absoluta*	% Adult emercence	Hb populations	***F***_**4,398**_ **= 19.722,** ***P** ***< 0.001**	*F*_4,398_ = 2.007, *P* = 0.093
		Sf populations	***F***_**4,398**_ **= 4.140,** ***P** ***= 0.003**	***F***_**4,399**_ **= 4.149,** ***P** ***= 0.003**
		Sc populations	***F***_**2,239**_ **= 17.490,** ***P** ***< 0.001**	*F*_2,239_ = 0.465, *P* = 0.629
	% Larval mortality	Hb populations	***F***_**4,443**_ **= 64.645,** ***P** ***< 0.001**	***F***_**4,439**_ **= 33.101,** ***P** ***< 0.001**
		Sf populations	***F***_**4,473**_ **= 1001.35,** ***P** ***< 0.001**	***F***_**4,439**_ **= 681.218,** ***P** ***< 0.001**
		Sc populations	***F***_**2,271**_ **= 243.946,** ***P** ***< 0.001**	***F***_**2,278**_ **= 133.477,** ***P** ***< 0.001**
	No. Days to kill	Hb populations	***F***_**4,327**_ **= 3.169,** ***P** ***= 0.014**	***F***_**4,476**_ **= 4.737,** ***P** ***< 0.001**
		Sf populations	***F***_**4,388**_ **= 40.353,** ***P** ***< 0.001**	***F***_**4,378**_ **= 22.344,** ***P** ***< 0.001**
		Sc populations	***F***_**2**,473_ = 0.917, *P* = 0.401	***F***_**2,178**_ **= 13.606,** ***P** ***< 0.001**

**Table S3.** Statistical analysis of the effect of the entomopathogenic nematodes (EPNs) infective juvenile (IJs) concentration (ANOVA, *P* < 0.05) for the variables percentage of adult emercence for *Frankliniella occidentalis* and *Tuta absoluta,* and the larval mortality percentage and number of days to kill the last instar larvae of *T. absoluta*. Analysis performed considering the control (C), and each of the populations of the three species *Heterorhabditis bacteriophora* (Hb)*, Steinernema feltiae* (Sf), and *S. carpocapsae* (Sc).

EPNs or populations	Insects	Variable	Comparative between concentrations				
*H. bacteriophora*			*C*	*Koppert*	*RM-102*	*AM-203*	*MG-618b*
	*F. occidentalis*	% Adult emercence	***t***_**188**_ **= 2.475,** ***P** ***= 0.016**	*t*_198_ = 0.153, *P* = 0.879	***t***_**198**_ **= 3.545,** ***P** ***= 0.001**	*t*_198_ = 1.929, *P* = 0.055	*t*_198_ = 1.845, *P* = 0.067
	*T. absoluta*	% Adult emercence	*t*_158_ = 1.856, *P* = 0.065	*t*_158_ = 1.105, *P* = 0.271	*t*_158_ = 1.426, *P* = 0.156	***t***_**157**_ **= 3.398,** ***P** ***= 0.001**	***t***_**157**_ **= 3.117,** ***P** ***= 0.002**
		% Larval mortality	*t*_182_ = 0.046, *P* = 0.963	***t***_**174**_ **= 2.383,** ***P** ***= 0.018**	***t***_**174**_ **= 2.766,** ***P** ***= 0.006**	*t*_174_ = 1.465, *P* = 0.145	*t*_174_ = 1.898, *P* = 0.060
		No. Days to kill	*t*_38_ = 0.103, *P* = 0.919	***t***_**111**_ **= 3.198,** ***P** ***= 0.002**	***t***_**139**_ **= 3.406,** ***P** ***= 0.002**	*t*_150_ = 1.431, *P* = 0.155	*t*_159_ = 0.272, *P* = 0.786
*S. feltiae*			*C*	*Koppert*	*RM-107*	*RS-5*	*AM-25*
	*F. occidentalis*	% Adult emercence	*t*_299_ = 0.028, *P* = 0.979	***t***_**198**_ **= 2.288,** ***P** ***= 0.023**	*t*_198_ = 0.880, *P* = 0.380	*t*_198_ = 0.721, *P* = 0.472	*t*_198_ = 0.423, *P* = 0.673
	*T. absoluta*	% Adult emercence	*t*_158_ = 0.532, *P* = 0.595	*t*_158_ = 0.157, *P* = 0.875	*t*_157_ = 0.566, *P* = 0.572	*t*_158_ = 0.476, *P* = 0.635	*t*_158_ = 1.432, *P* = 0.154
		% Larval mortality	*t*_185_ = 1.211, *P* = 0.289	No calcule* *P* = 1.0	***t***_**190**_ **= 2.032,** ***P** ***= 0.045**	***t***_**188**_ **= 2.011,** ***P** ***= 0.045**	No calcule* *P* = 1.0
		No. days to kill	*t*_10_ = 2.066, *P* = 0.066	*t*_189_ = 0.570, *P* = 0.569	***t***_**186**_ **= 4.723,** ***P** ***< 0.001**	***t***_**184**_ **= 2.882,** ***P** ***= 0.004**	***t***_**189**_ **= 2.043,** ***P** ***= 0.045**
*S. carpocapsae*			*C*	*Koppert*	*MG-596a*		
	*F. occidentalis*	% Adult emercence	*t*_216_ = 0.804, *P* = 0.422	***t***_**177**_ **= 3.692,** ***P** ***< 0.001**	*t*_198_ = 1.601, *P* = 0.111		
	*T. absoluta*	% Adult emercence	*t*_158_ = 0.532, *P* = 0.595	***t***_**158**_ **= 2.313,** ***P** ***= 0.022**	***t***_**158**_ **= 2.761,** ***P** ***= 0.006**		
		% Larval mortality	*t*_165_ = 1.445, *P* = 0.153	***t***_**190**_ **= 2.285,** ***P** ***= 0.025**	***t***_**190**_ **= 4.241,** ***P** ***< 0.001**		
		No. Days to kill	*t*_30_ = 0.210, *P* = 0.835	*t*_186_ = 0.169, *P* = 0.866	***t***_**166**_ **= 4.884,** ***P** ***< 0.001**		

Note: *The *t* student was not posible to estimate because there were not difference between the treatments (equal standard deviation).

### Virulence study against pupae of Tuta absoluta

**Figure 2:**
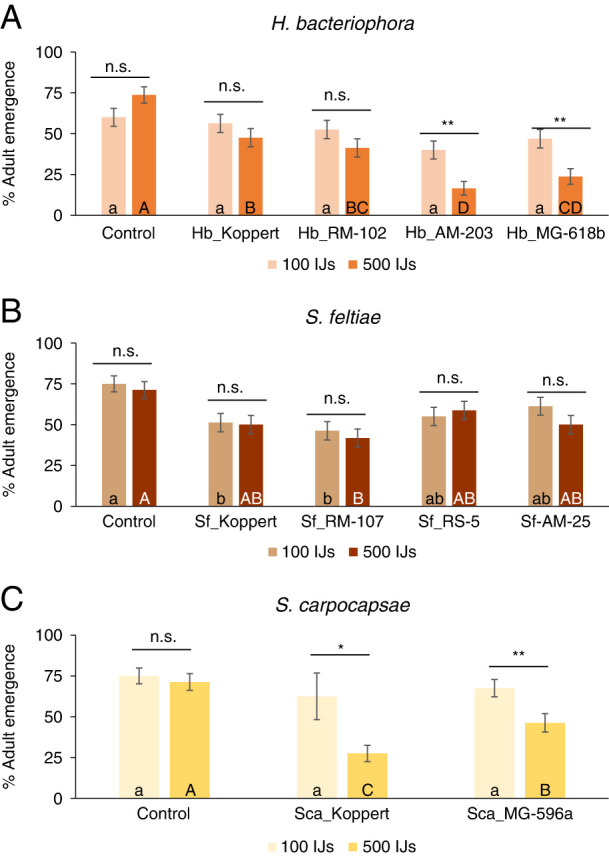
Intraspecific virulence of various populations from three entomopathogenic nematodes (EPN) species (*Steinernema feltiae*, *Steinernema carpocapase*, and *Heterorhabditis bacteriophora*) against pupa of *Tuta absoluta.* (**A**) Percentage of *T. absoluta* adult emergence after the application of four *H. bacteriophora* populations. (**B**) Percentage of *F. occidentalis* adult emergence after the application of four *S. feltiae* populations. (**C**) Percentage of *F. occidentalis* adult emergence after the application of two *S. carpocapsae* populations. Each population was applied in two concentrations: 500 JIs (21/cm^2^) and 100 JIs (4 IJs/cm^2^). Different small letters indicate significant differences in Tukey’s test (HSD) for the low concentration analysis. Different capital letters indicate significant differences in Tukey’s test (HSD). Above each species, asterisks indicate significant differences within treatment *t* student comparisons at *P < 0.05, **P < 0.01, ***P < 0.001, and n.s., not significant. Values are least-square means ± SE.

Percentage emergence of adults was significant for all the factors (populations and concentration) and their interactions except for *S. feltiae* (population and interaction), and the interactions in the intraspecific analysis for *S. carpocapsae* populations (Table S1, Fig. 2). We analyzed both factors individually to disentangle for concentration or population effects. Differences among populations were observed for the 500 IJs for all the species, but only among *S. feltiae* populations in the 100 IJs concentration (Table S2). In detail, in the analysis of *H. bacteriophora* populations, differences with the emergence observed in the control were only observed when the 500 IJs concentration was applied, registering values of 48-24% emergence (Fig. 2A). Differences between concentrations were observed for the populations Hb_AM-203 and Hb_MG-618b (Table S3, Fig. 2A), which showed values < 50% adult emergence even at the low concentration of 100 IJs. In the case of the *S. feltiae* populations, only Sf_Koppert resulted significantly lower than the control for both concentrations (Fig. 2B), and no population registered differences between concentrations (Table S3). Finally, for the *S. carpocapsae* intraspecific analysis, only the high concentration resulted significantly lower than the control emergence (Fig. 2C, Table S2), being the adult emergence of both populations significantly lower when applied at 500 IJs concentration (Fig. 2C, Table S3).

### Virulence study against last larval instar of Tuta absoluta

**Figure 3:**
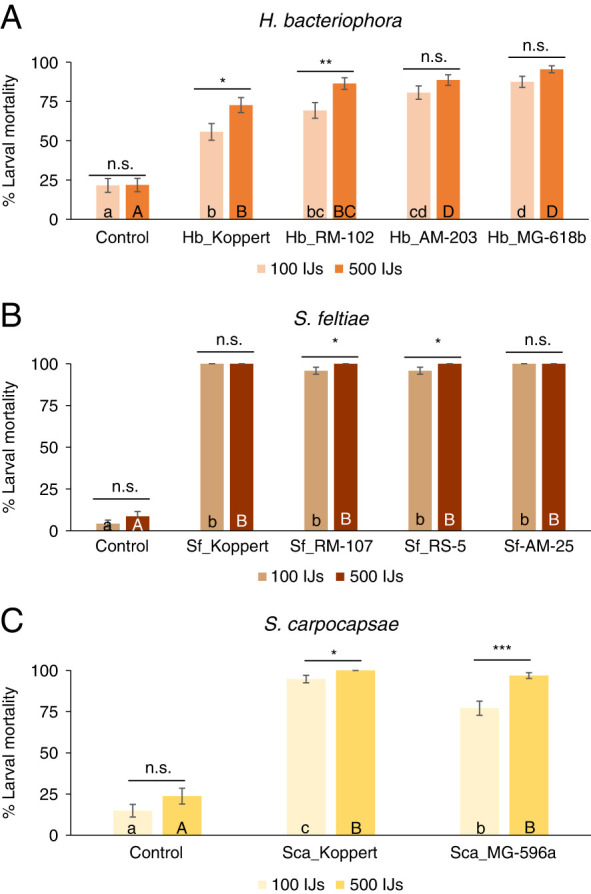
Intraspecific virulence of various populations from three entomopathogenic nematodes (EPN) species (*Steinernema feltiae*, *Steinernema carpocapase*, and *Heterorhabditis bacteriophora*) against last instar *Tuta absolua.* (**A**) Percentage of *T. absoluta* larval mortality after the application of four *H. bacteriophora* populations. (**B**) Percentage of *T. absoluta* larval mortality after the application of four *S. feltiae* populations. (**C**) Percentage of *T. absoluta* larval mortality after the application of two *S. carpocapsae* populations. Each population was applied in two concentrations: 500 JIs (21/cm^2^) and 100 JIs (4 IJs/cm^2^). Different small letters indicate significant differences in Tukey’s test (HSD) for the low concentration analysis. Different capital letters indicate significant differences in Tukey’s test (HSD). Above each species, asterisks indicate significant differences within treatment t student comparisons at *P < 0.05, **P < 0.01, ***P < 0.001, and n.s., not significant. Values are least-square means ± SE.

**Figure S1:**
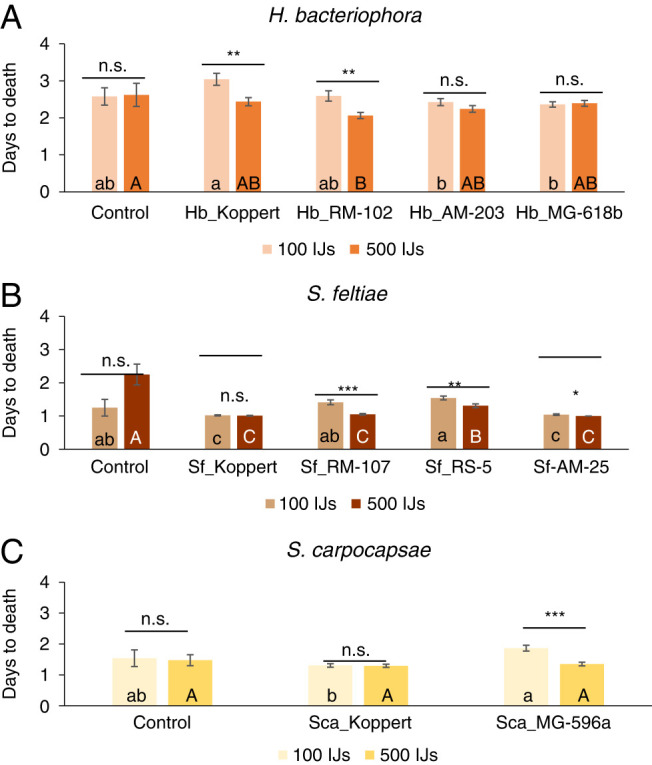
Intraspecific virulence of various populations from three entomopathogenic nematodes (EPN) species (*Steinernema feltiae, Steinernema carpocapase*, and *Heterorhabditis bacteriophora*) against last instar *Tuta absolua*. A. Number of days to kill *T. absoluta* larvae after the application of four *H. bacteriophora* populations. B. Number of days to kill *T. absoluta* larvae after the application of four *S. feltiae* populations. C. Number of days to kill *T. absoluta* larvae after the application of two *S. carpocapsae* populations. Each population was applied in two concentrations: 500 JIs (21/cm^^2^^) and 100 JIs (4 IJs/cm^^2^^). Different small letters indicate significant differences in Tukey’s test (HSD) for the low concentration analysis. Different capital letters indicate significant differences in Tukey’s test (HSD). Above each species, asterisks indicate significant differences within treatment t student comparisons at *P < 0.05, **P < 0.01, ***P < 0.001, and n.s., not significant. Values are least-square means ± SE.

Percentage larval mortality was significant for all the factors (populations and concentration), but not the interaction (Table S1, Fig. 3). When the number of days to kill the larva were considered, *H. bacteriophora* and *S. carpocapase* showed differences for both factors and the interactions (Table S1, Supplementary data Fig. S1). However, the intraspecific analysis of *S. feltiae* was not affected by concentration (Table S1, Fig. S1B). We analyzed both factors individually for each of the variables (larval mortality and time to kill) to disentangle for concentration or population effects. Differences among populations were observed in both concentrations for larval mortality percentage and number of days to kill the insect, except for the high concentration in *S. carpocapsae* (Table S2, Fig. 3, Fig S1). In detail, in the analysis of *H. bacteriophora*, all the populations reported significantly higher mortalities than the control for both concentrations (Fig. 3A), with differences between the two concentrations for Hb_Koppert and Hb_RM-102 (Table S3). Considering the time to kill the larva, only the population Hb_RM-102 killed faster than the mortality observed in the control treatments (Fig S1A). Differences between the concentrations were observed for Hb_Koppert and Hb_RM-102 (Table S3). In the case of the *S. feltiae*, all populations and both concentrations resulted different than the mortality observed in the control, ranging 95-100% (Fig. 3B, Table S2, Table S3). Differences between the concentrations were registered for Sf_RM-107 and Sf_RS-5. Considering the time to kill the larvae, all the populations were faster than control in the 500 IJs concentrations, while only Sf_Koppert and Sf_AM-25 did were at the low 100 IJs concentration (Fig. S1). All the populations were faster at the high concentration, except Sf_Koppert (Fig. S1, Table S3).Finally, for the *S. carpocapsae* intraspecific analysis, both populations and concentrations generated higher larval mortality than the control (Fig. 3C), but none resulted faster than control (Fig. S1). Higher IJs concentration resulted in higher mortality for both *S. carpocapsae* populations (Fig. 3C, Table S3), while only Sca_MG-596a resulted faster at the high concentration (Fig. S1, Table S3).

## Discussion

Overall, the species *S. feltiae* resulted in the most efficient in controlling both species using the low concentration, resulting *S. feltiae* Koppert population as the most consistent among the population tested. However, specific populations of the species *S. carpocapsae* and *H. bacteriophora* also registered high biocontrol efficiency against these two pests, highlighting the relevance of exploring the inter and intra-specific variability. The fact that *S.* feltiae was the best candidate among the EPN species tested is in agreement with several studies with both insect pests (Ebssa et al., 2001; van Damme et al., 2013; Gözel and Kasap, 2015). Indeed, populations of this species are commercialized to control *F. occidentalis* in greenhouses (Premachandra et al., 2003; Ebssa et al., 2004).

In agreement with previous studies, the three EPN species *S. feltiae, S. carpocapsae,* and *H. bacteriophora* were able to reduce the adult emergence of *F. occidentalis* and *T. absoluta* and kill the larvae of *T. absoluta* (Ebssa et al., 2001; Premachandra et al., 2003; Batalla-Carrera et al., 2010; van Damme et al., 2013; Gözel and Kasap, 2015). As expected, we observed differences in the virulence among the populations of each species. As a general pattern, whenever differences between the two concentrations were explored, the higher the IJs concentration applied, the lower resulted in the adult emergence, higher the larva mortality, and shorter the time to kill the insects.

Overall, the results observed against *F. occidentalis* are in agreement with the 30-50% mortality observed by Ebssa et al. (2001) when EPN were applied in concentrations ranging 100-200 IJs/cm^2^. Similarly, Dlamini et al. (2020) registered thrips mortality of ~50% when using *S. yirgalemense* concentrations up to 100 IJs/cm^2^. The efficiency of the EPN against *F. occidentalis* might depend on many factors. For example, ensuring warm temperatures and suitable moisture might enhance the action of the EPNs (Dlamini et al., 2020). Also, different species display a varying range of temperature and humidity viability (Grewal et al., 2006), so the selection of the most suitable EPN species accordingly the more frequently present environmental conditions will enhance their potential actions as biocontrol. Intraspecific variability has been also reported to modulate the EPN efficacy against *F. occidentalis* (Ebssa et al., 2001). It is likely that increasing the concentration of these species/populations might enhance their biocontrol action, as indicated by Ebssa et al. (2001). Also, to increase the biocontrol potential of the EPN, the timing and frequency of applications can be explored (Wardlow et al., 2001; Belay et al., 2005; Trdan et al., 2007). In addition, the combination of EPN with other biocontrol agents, such as the simultaneous release of predatory mites *Hypoaspis aculifer* and *Amblyseius cucumeris,* can result in higher control of *F. occidentalis* than individual applications (Premachandra et al., 2003; Ebssa et al., 2006). Finally, the symbiont bacteria might also play a role in the virulence of the EPN species and populations. Gerritsen et al. (2005) demostrated that toxin derived from the bacteria caused thrips mortality and reduce their fecundity. However, their screening of 46 *Photorhabdus* and six *Xenorhabdus* strains revealed inter and intraspecific variability on the thrips mortality, probably due to the differences in toxin production (Bode, 2009). Hence, when exploring the virulence of the EPN, attention shoul be also payed to the bacteria partner.

The three EPN species reduced the adult emergence of *T. absoluta* below 50% and generated > 85% larval mortality when applied at 21  IJs/cm^2^. The viability of these three EPN species against various instar of *T. absoluta* was previously observed (Batalla-Carrera et al., 2010; van Damme et al., 2013; Gözel and Kasap, 2015; Ben Husin and Port, 2021), and it is within the range to other EPN species such as *S. monticolum* (Shamseldean et al., 2014). In agreement with Batalla-Carrera et al. (2010), the pupa stage were less susceptible to *S. feltiae, S. carpocapsae,* and *H. bacteriophora* native populations. However, when these EPN were applied at 50 IJs/cm^2^ against pupae within the galleries, the mortality was < 10%. These values are lower than the 30-50% emergence observed in the present study. In contrast, the control produced in the larval stage resulted similar to those by Batalla-Carrera et al. (2010), 98-100% in the case of *S. feltiae,* resulting very efficiently considering that we employ 21 and 4 IJs/cm^2^. The later instar of *T. absoluta* is the most susceptible to the EPN (van Damme et al., 2013; Ben Husin and Port, 2021), and targetting this developmental stage might ensure the highest success. In agreement with previous studies, *S. feltiae* resulted in the most efficient EPN species in controlling *T. absoluta,* followed by *S. carpocapsae* (van Damme et al., 2013; Gözel and Kasap, 2015; Ben Husin and Port, 2021). Environmental conditions might modulate EPN efficiency. As described by the thrips, EPN species might differ in their range of temperature and humidity tolerance, extremes affecting survival, virulence, and reproduction ability (Grewal et al., 2006). Indeed, various studies reported differences in the EPN virulence depending on the environmental conditions and the EPN species (van Damme et al., 2013; Ben Husin and Port, 2021). For example, van Damme et al. (2013) observed that while *S. carpocapsae* and *H. bacteriophora* performed better at 25°C than at 18°C, *S. feltiae* produced 100% mortality in any condition. Similarly, Ben Husin and Port (2021) showed that while *S. feltiae* and *S. carpocapsae* resulted in equal virulence at 25°C, the species *S. feltiae* was more efficient at lower temperatures (15-20°C) while *S. carpocapsae* at higher temperatures (30-35°C). Our studies were performed at 22°C, a temperature that might be more conducive for the species *S. feltiae* than for *S. carpocapsae* and *H. bacteriophora.* Likely, changes in the temperature, like those described by van Damme et al. (2013) and Ben Husin and Port (2021), will modulate the efficiency observed for our populations. Similarly, relative humidity is critical to ensuring EPN search in the galleries (Ben Husin and Port, 2021). Hence, allowing a suitable environment perhaps linked to the specific requirement of a population can contribute to generate a successful control. About the intraspecific variability, overall, we observed that while the populations of *S. feltiae* resulted in equal efficiency, differences in *H. bacteriophora* and *S. carpocapase* populations were registered, mainly when applied at the low concentration of 4 IJs/cm^2^. It is plausible that *S. feltiae* might be better adapted to the search in the galleries, the environmental conditions, or both of those than the other species, where specific traits in one population might give an advantage.

The new application technologies such as the use of adyuvants and adapted agronomical machinery are expanding the use of EPN to new targets and crops that were unexplored before (i.e. vegetable and ornamental plantations, certain perennial systems such as vineyards) (Ben Husin and Port, 2021; Campos-Herrera et al., 2021). Selecting the best candidate is critical for promoting EPN use by growers. The screening should account not only for species but also different populations to ensure the viability of the selected species. This screening might serve to enhance certain traits by inbreeding with isolates of known performance (Bruno et al., 2020; Levy et al., 2020). This study highlighted the relevance of inter and intraspecific variability of the virulence, using two of the most critical aerial pests with worldwide distribution as the target. Global farming is evolving towards more personalized treatments, varying the strategies site-by-site. Studies on inter and intraspecific variability generate basic and applied knowledge to optimize the recommendation of use depending on the different ecological scenarios of each producer.
